# Effect of Feeding Adaptation of Italian Simmental Cows before Summer Grazing on Animal Behavior and Milk Characteristics

**DOI:** 10.3390/ani10050829

**Published:** 2020-05-11

**Authors:** Mirco Corazzin, Monica Berlese, Enrico Sturaro, Maurizio Ramanzin, Luigi Gallo, Eugenio Aprea, Flavia Gasperi, Damiano Gianelle, Stefano Bovolenta

**Affiliations:** 1 Department of Agricultural, Food, Environmental and Animal Sciences, University of Udine, 33100 Udine, Italy; monica.berlese@uniud.it (M.B.); stefano.bovolenta@uniud.it (S.B.); 2 Department of Agronomy, Food, Natural resources, Animals and Environment, University of Padova, 35020 Legnaro (PD), Italy; enrico.sturaro@unipd.it (E.S.); maurizio.ramanzin@unipd.it (M.R.); luigi.gallo@unipd.it (L.G.); 3 Research and Innovation Centre, Fondazione Edmund Mach, 38010 San Michele all’Adige (TN), Italy; eugenio.aprea@fmach.it (E.A.); flavia.gasperi@fmach.it (F.G.); damiano.gianelle@fmach.it (D.G.); 4 Center Agriculture Food Environment, University of Trento/Fondazione Edmund Mach, 38010 San Michele all’Adige (TN), Italy

**Keywords:** mountain, transhumance, feeding behavior, volatile compound, coagulation trait

## Abstract

**Simple Summary:**

The traditional transhumant system of rearing dairy cows in mountain areas expects animals to remain indoors in the valley during the cold season, whereas during the summer they are moved to pastures at progressively higher altitudes. The animals transferred from the valley farm to the alpine pasture must adapt to various management changes. This study aimed to evaluate whether a gradual inclusion of fresh grass in the diet of dairy cows in the valley farm can improve the performance and milk characteristics during summer grazing. Three groups of six animals each were considered: one group was kept in the stable, one was transferred from the valley to the summer farm without adaptation, and the other was progressively adapted to grazing with a feeding adaptation period. Compared to animals kept indoors, grazing animals had similar performance and milk characteristics, higher rumination time and, with respect to volatile compounds in milk, higher concentrations of alcohols, aldehydes, hydrocarbons, and ketones but lower concentrations of organic acids, phenolic compounds, and dimethyl sulfone, regardless of the feeding adaptation. In conclusion, the gradual inclusion of fresh grass in the diet in the valley farm did not improve the performance and milk characteristics during summer grazing.

**Abstract:**

According to the alpine transhumance system, dairy cows are moved from indoor feeding with conserved forage to fresh herbage feeding on pasture. The aim of this study was to assess, as a feeding adaptation technique, the effect of a gradual inclusion of fresh herbage in the diet of Italian Simmental dairy cows before their transfer to alpine pasture on performance, behavior, and milk characteristics. Eighteen cows were assigned to three groups: animals transferred to alpine pasture with a 10-d feeding adaptation period consisting in gradual access to a pasture close to the valley farm (GT), animals transferred to alpine pasture without a feeding adaptation period (AT), and animals kept in the valley farm (IND). During the first two weeks of summer grazing, GT and AT showed higher rumination time and different concentrations of ketones, hydrocarbons, organic acids, toluene, alcohols, phenols, and dimethyl sulfone in milk as compared to IND, whereas no differences were found in milk yield, composition, or coagulation properties. No differences between GT and AT were evident for the studied variables. The feeding adaptation technique used in this study did not influence the performance and milk characteristics of Italian Simmental dairy cows grazing on alpine pasture.

## 1. Introduction

Over the last few decades, mountain agricultural systems have undergone many economic, technological, and societal changes [1]. These changes have led to a reduction in the number of dairy farms, a significant increase in the average herd size, and a contraction of pasture areas. A general movement from grass-based to indoor systems has occurred, especially in valley areas [2,3]. However, mountain dairy farms are important for the manufacture of many products with specific features [4,5], for the environment [6], and for the provision of ecosystem services [7,8]. The traditional extensive system of rearing dairy cows in mountain areas is called “vertical transhumance” and consists of animals being kept in valley farms from autumn to early spring followed by the transfer of herds to high alpine pastures, gradually exploiting pastures at higher altitudes [9]. Nowadays, the system consists of dairy cows being moved from valley farms directly to high alpine pastures. In some areas of the Italian Alps, this practice may affect more than half of the farms [10]. Overall, during the first period of grazing, dairy cows have to face many stressful environmental and management changes, such as a different diet, hypoxia related to altitude, harsh climate conditions, increase in physical activity, change of milking system, and modification of social conditions [11,12]. Of these changes, one of the most important is the change in diet [13]. In fact, the animals are fed with a hay-based diet during the cold season and a fresh herbage-based diet during summer. This sudden change in feeding can cause many metabolic and physiological imbalances. In particular, the abrupt inclusion of fresh herbage in the diet alters the metabolism of fatty acids [14,15], favors the redistribution of energy towards maintenance requirements with a negative effect on milk production [16], influences dry matter intake [17] and milk composition [18], impairs the possibility to recover body reserves [19], and can even modify animal behavior [20]. Moreover, during the first few weeks after being moved from a total mixed ration (TMR) to fresh herbage, Schären et al. [21] observed a reduction in rumen fermentation activity in German Holstein cows, also leading to variations in the oxidation of dietary fatty acids and, therefore, in volatile organic compounds (VOCs) in milk [22].

Several studies have focused on the effects of summer grazing on milk characteristics [23,24] and milk VOCs [25,26]. In particular, summer grazing increases the fat content but decreases the protein content of milk [24], and it makes the fatty acid profile more favorable for human health [23]. Moreover, grazing can increase some milk VOCs such as terpenes [25], sulfur compounds, and alcohols [26]. Very scarce information is available on useful techniques to improve the feeding adaptation of animals to mountain grazing. Considering that the abrupt inclusion of grazing can negatively affect animal performance and alter rumen fermentation and milk characteristics, we hypothesize that a gradual inclusion of fresh herbage in the diet before the transfer to alpine pasture can favor the adaptation of dairy cows to alpine pasture in the short term. Furthermore, the experiment aimed to study the differences between grazing and indoor management in terms of performance, milk characteristics, and animal behavior.

## 2. Materials and Methods 

The study was conducted in accordance with EU Directive 2010/63/EU and Italian legislation (Legislative Decree no. 26, 4 March 2014), and adhered to the rules of the University of Udine. The ethical committee of the University of Udine was consulted. Since no invasive procedure was applied and the procedures adopted were routine, formal ethical approval was not required.

### 2.1. Experimental Design

Ten days before their transfer from the valley to the summer farm, 18 healthy Italian Simmental dairy cows with previous grazing experience belonging to the same farm (Tesero, TN, Italy, 1000 m above sea level) were randomly selected and assigned to three experimental groups: animals transferred to summer grazing with a feeding adaptation period (GT), animals transferred to summer grazing without a feeding adaptation period (AT), and animals kept in the valley farm throughout the experimental period (IND). The groups were homogeneous in terms of live weight (mean ± standard error (SE); 647 ± 18 kg), height at the withers (1.34 ± 0.08 m), lactation stage (195 ± 8 d in milk), number of lactations (1.9 ± 0.3), body condition score (BCS; 3.24 ± 0.07 points), milk yield (21.9 ± 1.1 kg/d of fat- and protein-corrected milk (FPCM)), and milk composition (70,020 ± 14,650 cells/mL somatic cell count (SCC); 3.45% ± 0.04% protein, 3.64% ± 0.09% fat, 2.69% ± 0.04% casein). The experimental period lasted 24 d, 10 d before and 14 d after the transfer of animals (groups GT and AT) to the summer farm (Malga Juribello, Paneveggio Pale San Martino Natural Park, TN, lat. 46°29’66’’, long. 11°78’67’’, 1860 m above sea level). Before the start of the experimental period, all animals were indoor housed in a cubicle system and were fed ad libitum with the same TMR (dry matter (DM) basis) composed of 25% grass silage, 25% grass hay, and 50% concentrate (maize meal, wheat meal, whole soybean, barley meal, soybean meal, molasses, vitamins, and minerals). This diet was maintained throughout the experimental period for the IND group. Before summer grazing, the same diet was also offered ad libitum to the GT and AT groups. However, with the aim of improving the feeding adaptation to summer grazing, the GT group was still fed ad libitum with the same TMR but also gradually introduced to a pasture-based diet. In particular, GT had access to a pasture close to the farm, less than 50 m in walking distance, for 1 h/d, days 9–10; 3 h/d, days 7–8; 5 h/d, days 5–6; 7 h/d, days 3–4; 9 h/d, days 1–2 before the transfer to the summer farm. The pasture was an *Arrhenatherion elatioris* W. Koch 1926 alliance mainly composed of *Arrhenatherum elatius*, *Poa pratensis*, *Trisetum flavescens*, *Dactylis glomerata,* and *Alopecurus pratensis*. Transportation by truck from the valley to the summer farm (including loading and unloading) lasted 90 min. Dairy cows in the GT and AT groups grazed together in a herd of 140 cows in a 19-ha alpine pasture under a shepherd-guided grazing system. The alpine pasture was a *Poion alpinae* Oberd. 1950 alliance mainly composed of *Poa alpina*, *Phleum alpinum*, *Trifolium pratense*, *Trifolium repens*, *Alchemilla vulgaris,* and *Carum carvi*. During summer grazing, GT and AT received 5 kg DM of a concentrate composed of wheat bran, corn, sugar beet pulps, sunflower, molasses, vitamins, and minerals. The concentrate was fed in twice-daily meals during milking. The animals were kept day and night on pasture. Samples of TMR, pasture and concentrate were collected every 3 days, dried at 65 °C in a forced draft oven for 48 h, and analyzed for crude protein (CP) and ether extract (EE) following Association of Official Agricultural Chemists [27], and for neutral detergent fiber (NDF) following Goering and Van Soest [28]. The diets were formulated and the energetic values of feed were assessed according to Baumont et al. [29]. Furthermore, the energetic value of feed was expressed as net energy for lactation (NEL). Considering TMR, the hay had 12.2% DM CP, 60.3% NDF, and 4.6 MJ NEL/kg DM; the herbage silage had 14.3% DM CP, 57.5% NDF, and 4.3 MJ NEL/kg DM; and the concentrate had 15.5% DM CP and 8.1 MJ NEL/kg DM. The pasture close to the farm had 15.1% DM CP, 69.9% NDF, and 5.1 MJ NEL/kg DM; the alpine pasture had 15.2% DM CP, 56.8% NDF, and 5.2 MJ NEL/kg DM; the concentrate offered to animals in the alpine farm had 12.4% DM CP and 7.0 MJ NEL/kg DM.

### 2.2. Measurements

Environmental temperature and rainfall were recorded by two public automatic weather stations located near the valley and summer farms throughout the experimental period. BCS of animals was assessed 17 and 24 d (weekly during grazing) after the beginning of the experiment, following Edmonson et al. [30]. Throughout the experimental period, the dairy cows were equipped with a noseband pressure sensor and a pedometer (RumiWatch system, ITIN-HOCH GmbH, Liestal, Switzerland) for assessing the feeding and locomotion behavior, as reported by Romanzin et al. [31]. The variables recorded were eating and rumination time (min/d); eating and rumination chews (n/d); number of rumination boli (n/d); lying, walking and standing time (min/d); number of steps (n/d). The feeding and locomotion behavior recorded 10 d after the beginning of the experiment (during the grazing period), excluding milking time, was considered for analyses.

### 2.3. Sampling and Analysis

Individual milk yield was recorded and samples were collected 14, 17, 21, and 24 d after the beginning of the experiment, considering both evening and morning milking. Milk samples were refrigerated without preservative for milk coagulation properties (MCP) and VOCs, and with preservative for the other analyses. In particular, milk samples were analyzed for fat, protein, lactose, casein, urea, β-hydroxybutyrate (BHB), and acetone using MilkoScan FT6000 (FOSS Electric, Hillerød, Denmark) and calibrated according to the International Dairy Federation Standard [32]; for somatic cell count (SCC) using Foss-o-Matic (FOSS Electric, Hillerød, Denmark) and calibrated according to the International Dairy Federation Standard [33]; and for coagulation properties (MCP). The analysis of MCP was based on the 9-MilCA method, which mimics dairy cheese making, as reported by Cipolat Gotet et al. [34]. Individual morning and evening milk samples were analyzed separately, and the average values weighted by the corresponding milk yield were considered for statistical analysis. Milk yield was analyzed as FPCM [35]. At each sampling time, but considering only morning milking, VOCs were measured by solid-phase microextraction (SPME) GC-MS. This method has been optimized previously [36]. Briefly, 5 mL of milk was poured into a 20 mL glass vial with 4-methyl-2-pentanone used as an internal standard. VOCs were extracted at 40 °C with a Divinylbenzene-Carboxen–Polydimethylsiloxane SPME (2 cm length) and then desorbed in the injector port (250 °C) of the GC interfaced with a mass detector (electron ionization; internal ionization source; 70 eV) scanning from m/z 33 to m/z 300 (GC Clarus 500, PerkinElmer, Norwalk, CT). SPME analysis was automated by an auto-sampling system (CTC combiPAL, CTC Analysis AG, Zwingen, Switzerland). Separation was achieved on a HP-Innowax fused-silica capillary column (30 m, 0.32 mm inner diameter, 0.5 μm film thickness; Agilent Technologies, Palo Alto, CA). The temperature program was set as follows: 40 °C for 3 min, 180 °C for 6 min at 4 °C min^−1^, 220 °C for 3 min at 3 °C min^−1^. Helium at a flow rate of 2 mL/min was used as a carrier gas. The transfer line temperature was kept at 220 °C. Linear retention indices (LRI) were calculated under the same chromatographic conditions, injecting C7-C30 n-alkane series (Supelco, Bellefonte, PA). Compounds were identified by using the mass spectra matching the National Institute of Standards and Technology-2014/Wiley 7.0 libraries and comparing the calculated LRI with those available from the literature. 

### 2.4. Statistical Analysis

The statistical analyses were performed using R software, version 3.4.0 [37]. The normality and homoscedasticity of data were tested using Shapiro–Wilk and Levene tests, respectively. When appropriate, variables were transformed for parametric testing. The effects of the adaptation method (GT, AT, IND) on the variables related to milk characteristics during summer grazing were assessed with a mixed model for repeated measures, as suggested by Wang and Goonewardene [38], considering the adaptation method as a fixed factor and the day of sampling as a repeated factor. Individual animals were treated as a random factor. The interaction of adaptation method × day of sampling was also considered. If this interaction was significant, the differences between adaptation methods for the specific day of sampling were evaluated, following Park et al. [39]. Fisher’s least significant difference was used as a post-hoc test. The same model was considered for BCS and for the variables related to feeding and locomotion behavior, but instead of the day of sampling, the day of assessment and the day of measurement were considered, respectively. VOCs were also subjected to principal components analysis (PCA) using SIMCA-P+12.0 (Umetrics, Umea, Sweden). Data were log-transformed and scaled to unit variance before PCA.

In the manuscript, values are reported as mean ± SE. Differences were considered significant at *p* ≤ 0.05.

## 3. Results and Discussion

### 3.1. Weather Conditions

During grazing time, the average temperature was 9.6 °C (min. 5.9 °C, max. 13.1 °C); the median precipitation was 4.0 mm/d (min. 0 mm/d, max. 30 mm/d). There were 11 rainy days, but the precipitation was more than 5.0 mm on just 3 days. During the same time, in the valley farm the average temperature was 17.2 °C (min. 13.7 °C, max. 19.5 °C), the median precipitation was 0 mm/d (min. 0 mm/d, max. 23 mm/d; data not reported in tables). The average sunrise was at 5:21 a.m., and the average sunset was at 9:07 p.m., giving an average length of day of approximatively 16 h. Since all the experimental groups were kept within the thermo-neutral zone for dairy cattle [40], the productive performance can be considered not affected by the environmental conditions.

### 3.2. Body Conditions Score and Milk Yield, Composition, and Coagulation Properties

Table 1 reports the results for BCS and milk yield and composition. No effect of adaptation method on BCS or milk yield was found (*p* > 0.05). However, the interaction of adaptation method × day of assessment was significant for BCS. In the IND group, the BCS increased from 17 to 24 d (3.20 ± 0.12 points vs. 3.27 ± 0.10 points; *p* < 0.05); conversely, the BCS of GT (3.19 ± 0.13 points vs. 3.10 ± 0.11 points; *p* > 0.05) and of AT (3.33 ± 0.06 points vs. 3.29 ± 0.05 points; *p* > 0.05) remained constant. The animals were in late lactation, so an increase in BCS should be expected. However, several studies have shown that it is difficult for dairy cows to restore their body reserves during summer grazing on alpine pasture [41,42,43] due to the high energy expenditure of their physical activity. Furthermore, the length of the grazing period in this experiment may not be enough to observe variations in animal BCS. Considering milk composition, fat, protein, lactose, urea, SCC, and casein were not affected by adaptation method (*p* > 0.05). However, for fat, casein, and urea, a significant interaction of adaptation method × day of sampling was found (*p* < 0.05). AT had higher fat content in milk than IND only at 21 d after the beginning of the experiment (4.42% vs. 3.68%; *p* < 0.05) while GT showed intermediate values (4.18%). Grazing animals (GT and AT) had higher casein in their milk than IND animals only at the second sampling time (3.95%, 3.88%, and 3.22% for GT, AT, and IND, respectively; *p* < 0.05). Considering urea, 4 days after the beginning of summer grazing (first sampling time), IND showed the lowest value (15.04 ± 2.25 mg/dL; *p* < 0.01) while GT had a lower value than AT (24.38 ± 0.93 vs. 28.86 ± 1.95 mg/dL; *p* = 0.09). Within the subsequent samplings, no differences between the experimental groups were detected (*p* > 0.05). Milk urea increases with increasing dietary protein-to-energy ratio; therefore, the higher values observed in grazing animals could be due to the higher level of fermentable protein that characterizes the pasture at the beginning of the grazing season [44]. However, the fact that GT tended to have a lower level of milk urea than AT during the first few days of the upland grazing season could be due to rumen micro-organisms that adapted faster to the new feeding. In general, all the experimental groups showed urea contents in milk within the range of normality proposed by Bendelja et al. [45], i.e., 15–30 mg/dL. The contents of acetone and BHB were affected by adaptation method (*p* < 0.05), but also the interaction of adaptation method × day of sampling was significant (*p* < 0.05). Both acetone and BHB are indicators of ketosis and of a negative energy balance in cows if their concentration in milk exceeds 0.70 and 0.15 mmol/L, respectively [46,47]. The results indicated that the animals in the present study were not in a state of negative energy balance.

As shown in Table 1, the MCP were not affected by adaptation method (*p* > 0.05), and the average values fall within the ranges proposed by Cecchinato et al. [48]. Many studies have reported that dairy cows grazing on mountain pasture produce milk with impaired MCP [49,50]. The reasons for this may be a high NDF and/or low protein content in forage, and in general, a low level of protein in milk that could be related to the possible negative energy balance of dairy cows during grazing [12]. In fact, Bovolenta et al. [51] observed an improvement in milk coagulation time and curd firmness when the supplement offered to grazing animals was increased from 1.8 to 5.3 kg DM/day. As previously discussed, the energy balance condition of the animals and the similar protein contents in milk can explain the lack of significant differences in MCP observed in the present experiment. Additionally, Saha et al. [24] observed similar MCP in milk produced from indoor-fed cows to that produced from grazing cows supplemented with 5 kg/d of concentrate.

The few differences observed between experimental groups could be partially due to animal breed. Indeed, genotype can affect the effectiveness of animal responses to environmental changes [52], and Italian Simmental is a dual-purpose breed that is spreading in the Alpine area thanks to its good adaptability to mountain grazing conditions [53].

### 3.3. Milk Volatiles Organic Compounds

In Table 2, the VOCs in milk are reported. A total of 50 VOCs belonging to alcohols (8), aldehydes (8), hydrocarbons (10), ketones (6), organic acids (11), phenolic compounds (3), terpenes (1), sulfur compounds (1), and lactones (2) were identified. Toso et al. [54] and Villeneuve et al. [26] reported 41 and 50 volatile compounds, respectively, in the milk of dairy cows fed with hay and silage-based diets. The sum of alcohols (mainly due to 1-pentanol, 1-hexanol, and 1-heptanol concentrations) and the sum of hydrocarbons (mainly due to toluene, limonene, and dimethyl sulfone concentrations) were higher in milk from grazing animals than in milk from the IND group (*p* < 0.05). However, for limonene and for the sum of hydrocarbons, the interaction of adaptation method × day of sampling was significant (*p* < 0.05). The sum of aldehydes was higher in the AT than the IND group (*p* < 0.05) with intermediate values shown in GT, reflecting the results for hexanal concentrations. The sum of ketones as well as the concentration of 2-pentanone were higher in milk from grazing animals than in milk from the IND group (*p* < 0.05). Conversely, the sum of organic acids (mainly due to butanoic, hexanoic, octanoic, and decanoic concentrations) and phenolic compounds were lower in milk from grazing animals than in milk from the IND group (*p* < 0.05). No effect of adaptation method on lactones was found (*p* > 0.05). Bergamaschi and Bittante [55] highlighted that volatile compounds can derive from the degradation of fat and protein by enzymes naturally present in milk and associated with the presence of somatic cells. However, as previously reported, in the present experiment the SCC was similar between experimental groups. More importantly, milk volatile compounds can also derive directly from the diet of the animals; from the metabolism of carotenoids, amino acids, and carbohydrates; and from the oxidation of unsaturated fatty acids (UFA) in milk [22]. Herbage is particularly rich in UFA such as linolenic acid [23]. Although ruminal micro-organisms saturate part of the dietary UFA, milk from grazing cows is richer in polyunsaturated fatty acids (PUFA) [56]. Therefore, milk from grazing animals is more susceptible to oxidation [22]. However, the extent of this biohydrogenation as well as the metabolism of carotenoids, amino acids, and carbohydrates depend on rumen conditions, particularly different bacterial populations [57]. In agreement with the results obtained for milk characteristics and animal feeding behavior, the milk VOCs seem more related to the forage offered to animals, herbage vs. silage/hay, rather than to the different rumen condition that could be derived from the feeding adaptation technique adopted, GT vs. AT. Indeed, it is interesting to note that 1-pentanol and 1-hexanol, which derive from fatty acid oxidation [22] were highest in the milk of grazing animals; conversely, the concentration of 3-methyl-1-butanol, which derives from amino acid metabolism in the rumen, was similar between experimental groups. Villeneuve et al. [26] found significantly or numerically higher concentrations of 1-pentanol, 1-hexanol, and 1-heptanol in the milk of cows fed with pasture than in the milk of those fed with timothy hay. The sum of aldehydes was higher in AT than IND, and GT showed a similar value to IND, reflecting the result of hexanal concentration. Conversely, heptanal was higher in the milk of grazing animals than in the milk of IND animals. Aldehydes derive mainly from lipid oxidation. Hexanal is a product of the oxidation of oleic acid and linoleic acid, which are also present in concentrates; it is a transient product and can be converted to 1-hexanol [22]. Toluene concentration was higher in milk from grazing animals. Since it derives from the degradation of *β*-carotene in the rumen, toluene is considered a marker of the use of pasture by animals [22], as also shown by Villeneuve et al. [26]. Therefore, the results of the present study reflect the higher fresh herbage intake in grazing animals. A similar hypothesis can be drawn for the higher content of ketones observed in grazing animals. Indeed, ketones derive mainly from the oxidation of dietary PUFA. The content of butanoic and hexanoic acids was lower in milk from grazing animals than in milk from IND animals. These organic acids derive from lipolysis and de-novo synthesis in the mammary gland [22], which is reduced by high dietary levels of UFA and linolenic acid in particular. The results of the present experiment are in agreement with the findings of Lee et al. [58], who observed a reduction in short chain fatty acids in milk obtained from grazing animals with respect to milk obtained from indoor-fed animals. In the present experiment, dimethyl sulfone was higher in the milk of grazing animals than in that of IND animals. Dimethyl sulfone derives from the metabolism of methionine in the rumen [22]. In agreement with this result, Coppa et al. [25] also observed a higher content of dimethyl sulfone in the milk of cows fed with pasture-based diets than in that produced by animals fed with hay-based diets. The above-cited authors explain that fresh herbage has a higher methionine content and a higher protein-to-readily digestible carbohydrates ratio than dry forage. Conversely, the level of dimethyl sulfone was similar in milk from GT and AT, further confirming the hypothesis that the rumen conditions were similar between these groups. In general, the results of this study showed that the milk VOCs were influenced little by the feeding adaptation technique adopted.

**Figure 1:**
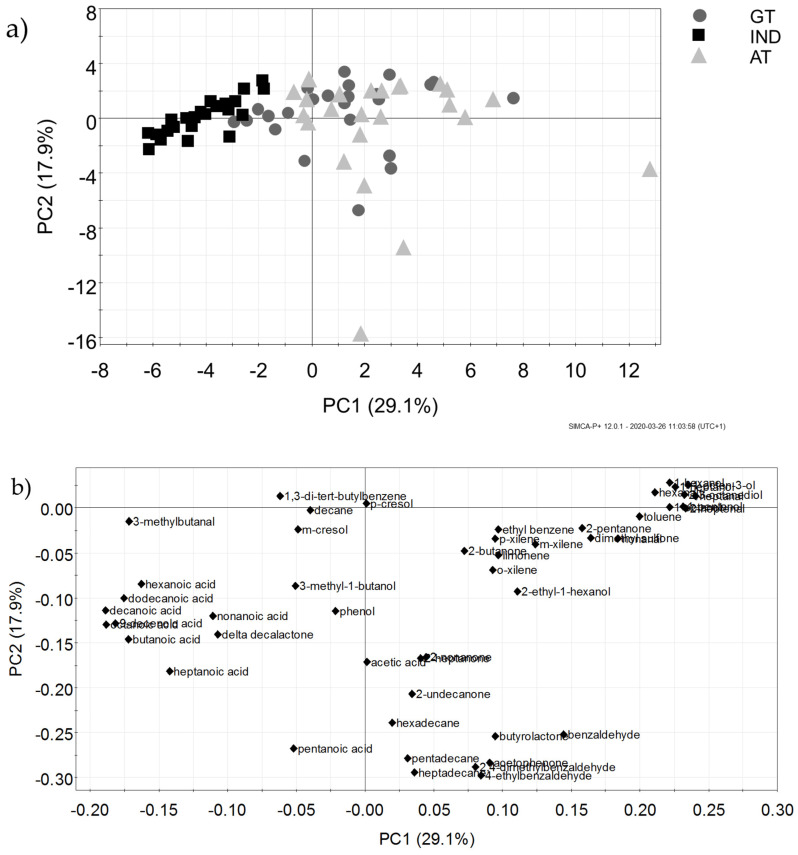
Principal component analysis of volatile organic compounds (VOCs) showing experimental groups’ milks (**a**); GT = dairy cows adapted to fresh herbage feeding before grazing; AT = dairy cows not adapted to fresh herbage feeding before grazing; IND = dairy cows kept in a cubicle system) and individual VOCs (**b**).

Figure 1 shows an overall illustration of VOCs in experimental milk. The first and second principal components (PCs) explained 29.1% and 17.9% of the total variance, respectively. The IND group’s milk was separated on the first PC from those of GT and AT (Figure 1a) and was mainly positively related to organic acids (Figure 1b). Milk from the GT and AT groups could not be clearly separated (Figure 1a), and was mainly positively related to alcohols, aldehydes, and toluene (Figure 1b). It is also interesting to note that variability in milk volatile compounds is higher for the AT group (Figure 1a), mainly because of the higher amount of aromatic hydrocarbons.

### 3.4. Feeding and Locomotion Behavior

The feeding behavior of animals is reported in Table 3. No effect of adaptation method on the number of eating chews was found (*p* > 0.05). However, although a significant level was not achieved, eating time was numerically higher in AT and GT than in IND. Beauchemin [59] reported that eating time increases with increasing dietary particle size. Grazing animals had lower rumination time (*p* < 0.01), rumination chews (*p* < 0.01), and number of boli per day (*p* < 0.05) than IND. Ruminating time depends on many factors. Beauchemin [59] explained that, for low-yielding cows, a compensatory mechanism between ruminating and eating times can be observed; in other words, the more time is spent eating, the less time is spent ruminating. Moreover, the shorter ruminating time observed in grazing animals could be due to the higher degradability of fresh forage than that of indoor feed. This hypothesis could also contribute to explaining the lower number of rumination chews and number of boli per day highlighted in grazing animals. Ocak [60] reported a higher rate of degradation in the rumen for fresh forage than for dry forage. It is well known that rumination times are linked to correct ruminal functionality. In the present study, the average ruminating time of IND was very similar to that reported by De Vries et al. [61], 555 min/d, in healthy cows fed with a 60:40 forage-to-concentrate ratio. The ruminating times observed in grazing animals were lower than those reported by Romanzin et al. [31], 473 min/d, but within the range reviewed by Braun et al. [62], 240–584 min/d. The locomotion behavior of animals is reported in Table 3. As expected, grazing animals showed much higher walking time and number of steps per day than IND (*p* < 0.05). For these variables, the interaction of adaptation method × day of measurement was also significant (*p* < 0.01) but ordinal from the perspective of the adaptation method factor. This means that grazing animals had a higher walking time and number of steps than indoor-housed animals per day of measurement. Thus, the effect of adaptation method can be considered separately from the day of measurement effect [63]. Conversely, no effect of adaptation method on standing time was found (*p* > 0.05).

## 4. Conclusions

Since many farmers report negative effects of the direct transfer of dairy cows from indoor housing in the valley to summer farms, this study aimed to verify possible beneficial effects of a feeding adaptation before summer grazing. In the present experiment, in which the Italian Simmental dual-purpose breed was used, grazing animals showed similar performance and milk characteristics but different feeding behavior and milk VOCs compared to animals kept indoors in the valley farm in the short term, regardless of feeding adaptation. Indeed, during the first two weeks, grazing animals showed higher rumination time and number of rumination chews and boli, and different concentrations of ketones, hydrocarbons, organic acids, toluene, alcohols, phenols, and dimethyl sulfone in their milk as compared to indoor-housed animals. The observed differences seemed to be more related to the fresh herbage intake rather than to the feeding adaptation technique used. Further studies involving longer adaptation periods, high-producing breeds, and different stages of lactation are needed.

## Figures and Tables

**Table 1:** Mean values of body condition score (BCS) and milk yield, composition, and coagulation properties (MCP) (*n* = 18).

Item	Adaptation Method			SEM	*p*-Value		
	GT	AT	IND		AM	D	AM × D
BCS (points)	3.15	3.31	3.23	0.058	0.52	0.41	0.02
FPCM (kg)	18.9	22.2	22.9	1.23	0.78	0.39	0.16
Fat (%)	4.00	4.07	3.77	0.084	0.36	0.01	0.01
Protein (%)	3.54	3.52	3.52	0.032	0.94	<0.01	0.05
Lactose (%)	4.70	4.69	4.70	0.052	0.14	0.49	0.36
Urea (mg/dL)	16.98	19.23	17.79	0.714	0.43	<0.01	<0.01
SCC (×1000 cells/mL)	90.4	109.8	206.6	57.06	0.79	0.05	0.49
Casein (%)	2.82	2.80	2.73	0.027	0.39	<0.01	<0.01
Acetone (mmol/L)	0.021 ^b^	0.033 ^ab^	0.057 ^a^	0.006	0.04	<0.01	<0.01
BHB (mmol/L)	0.039 ^b^	0.051 ^b^	0.092 ^a^	0.006	<0.01	<0.01	0.02
RCT (min)	31.95	24.03	24.15	1.857	0.17	0.80	0.01
k_20_ (min)	8.36	5.94	7.02	0.612	0.30	0.05	0.21
a_45_ (mm)	26.01	34.59	30.60	2.770	0.47	<0.01	0.38
a_60_ (mm)	30.26	35.11	32.89	1.510	0.44	<0.01	0.76

GT = dairy cows adapted to fresh herbage feeding before grazing; AT = dairy cows not adapted to fresh herbage feeding before grazing; IND = dairy cows kept in cubicle system; SEM = standard error of the mean; AM = adaptation method; D = day of assessment for BCS-sampling day for milk yield and composition; FPCM = fat and protein corrected milk; SCC= somatic cells count; BHB: *β*-hydroxybutyrate; RCT = rennet coagulation time; k 20 = time from milk gelation to 20 mm of curd firmness equivalent; a 45, a60 = curd firmness recorded after 45 and 60 min from rennet addition; ^a,b^: means statistically different.

**Table 2:** Mean values of volatiles organic compounds (μg/kg equivalent of 4-methyl-2-pentanone [36]) in milk (*n* = 18).

Volatile Organic Compound	Adaptation Method			SEM	*p*-Value		
	GT	AT	IND		AM	D	AM × D
Alcohols							
3-methyl-1-butanol	1.55	2.06	2.73	0.580	0.80	0.04	0.23
1-pentanol	4.84 ^a^	5.37 ^a^	0.81 ^b^	0.352	<0.01	0.16	0.23
2,3-octanediol	0.45 ^a^	0.91 ^a^	0.07 ^b^	0.089	<0.01	0.12	0.18
1-hexanol	7.21 ^a^	8.80 ^a^	0.91 ^b^	1.322	<0.01	0.31	0.62
1-octen-3-ol	0.47 ^a^	0.62 ^a^	0.09 ^b^	0.046	<0.01	0.18	0.25
1-heptanol	0.85 ^a^	1.14 ^a^	0.04 ^b^	0.152	<0.01	0.21	0.44
2-ethyl-1-hexanol	0.86 ^ab^	1.07 ^a^	0.68 ^b^	0.042	0.01	<0.01	<0.01
1-octanol	0.25 ^a^	0.39 ^a^	0.03 ^b^	0.037	<0.01	0.01	0.19
Sum, alcohols	16.48 ^a^	20.36 ^a^	5.36 ^b^	1.798	<0.01	0.13	0.52
Aldehydes							
3-methylbutanal	1.07 ^b^	0.42 ^b^	4.73 ^a^	0.348	<0.01	0.34	0.26
hexanal	17.47 ^ab^	35.14 ^a^	5.81 ^b^	3.354	0.02	0.48	0.34
heptanal	2.74 ^a^	6.68 ^a^	0.21 ^b^	0.712	<0.01	0.34	0.13
(E)-2-heptenal	0.15 ^a^	0.27 ^a^	0.01 ^b^	0.026	<0.01	0.29	0.58
nonanal	0.67 ^ab^	1.01 ^a^	0.34 ^b^	0.077	0.01	<0.01	0.54
benzaldehyde	0.37 ^ab^	0.53 ^a^	0.24 ^b^	0.029	0.01	0.06	0.26
4-ethylbenzaldehyde	0.02 ^ab^	0.03 ^a^	<0.01 ^b^	0.004	0.03	0.38	0.44
2,4-dimethylbenzaldehyde	0.01	0.02	<0.01	0.004	0.07	0.45	0.22
Sum, aldehydes	22.50 ^ab^	44.10 ^a^	11.38 ^b^	4.038	0.04	0.25	0.66
Hydrocarbons							
decane	1.95	2.09	2.47	0.451	0.16	<0.01	<0.01
toluene	26.30 ^a^	29.40 ^a^	0.55 ^b^	1.808	<0.01	<0.01	0.07
ethyl benzene	0.19 ^b^	0.85 ^a^	0.33 ^b^	0.133	0.01	0.59	0.18
*p*-xilene	0.08	0.26	0.13	0.042	0.07	0.81	0.11
*m*-xilene	0.15 ^b^	0.38 ^a^	0.17 ^b^	0.054	0.01	0.45	0.08
*o*-xilene	0.15 ^b^	0.30 ^a^	0.20 ^a^	0.035	0.03	<0.01	<0.01
1,3-di-tert-butylbenzene	0.98 ^b^	1.01 ^ab^	1.63 ^a^	0.064	<0.01	<0.01	<0.01
pentadecane	0.06	0.09	0.02	0.021	0.42	0.14	0.26
hexadecane	0.05	0.10	0.05	0.008	0.44	0.36	0.69
heptadecane	0.04	0.06	0.03	0.008	0.60	0.62	0.22
Sum, hydrocarbons	29.95 ^a^	34.53 ^a^	5.61 ^b^	5.174	<0.01	<0.01	<0.01
Ketones							
2-butanone	2.11	2.13	1.68	0.131	0.29	0.38	0.62
2-pentanone	1.51 ^a^	1.79 ^a^	1.02 ^b^	0.111	0.01	0.71	0.08
2-heptanone	4.48	6.27	1.04	1.412	0.49	0.75	0.95
2-nonanone	3.17	4.90	0.26	1.214	0.35	0.67	0.88
2-undecanone	0.03	0.04	0.02	0.009	0.52	0.95	0.56
acetophenone	0.10	0.13	0.06	0.011	0.30	0.76	0.92
Sum, ketones	11.40 ^a^	15.26 ^a^	4.08 ^b^	2.660	0.05	0.74	0.70
Organic acids							
acetic acid	0.24	0.19	0.15	0.020	0.22	0.62	0.20
butanoic acid	2.54 ^b^	2.95 ^b^	6.35 ^a^	0.460	0.01	0.73	0.97
pentanoic acid	0.05	0.05	0.05	0.008	0.67	0.09	0.13
hexanoic acid	5.44 ^b^	6.75 ^b^	14.80 ^a^	1.179	0.01	0.66	0.76
4-methyl hexanoic acid	0.01	<0.01	<0.01	0.005	0.43	0.37	0.43
heptanoic acid	0.13 ^b^	0.15 ^b^	0.26 ^a^	0.014	0.01	0.47	0.46
octanoic acid	4.47 ^b^	5.14 ^b^	13.18 ^a^	0.828	<0.01	0.39	0.94
nonanoic acid	0.65 ^b^	0.62 ^b^	1.12 ^a^	0.077	0.05	0.68	0.69
decanoic acid	2.45 ^b^	2.53 ^b^	6.11 ^a^	1.230	<0.01	0.14	0.41
9-decenoic acid	0.10 ^b^	0.12 ^b^	0.30 ^a^	0.019	<0.01	0.41	0.73
dodecanoic acid	0.34 ^b^	0.32 ^b^	0.63 ^a^	0.037	0.01	0.75	0.28
Sum, organic acids	16.41 ^b^	18.82 ^b^	42.94 ^a^	9.102	<0.01	0.15	0.51
Phenolic compounds							
phenol	0.37 ^b^	0.37 ^b^	0.42 ^a^	0.008	<0.01	1.00	0.92
*p*-cresol	0.23	0.21	0.25	0.008	0.14	0.67	0.99
*m*-cresol	0.69 ^b^	0.63 ^b^	0.83 ^a^	0.023	<0.01	0.70	0.98
Sum, phenolic compounds	1.28 ^b^	1.20 ^b^	1.49 ^a^	0.036	0.01	0.80	0.97
Terpene							
limonene	0.41 ^a^	0.41 ^a^	0.01 ^b^	0.051	0.01	<0.01	0.01
Sulphur compound							
dimethyl sulfone	3.41 ^a^	4.83 ^a^	1.91 ^b^	0.230	<0.01	0.23	0.12
Lactones							
γ-butyrolactone	0.36	0.46	0.25	0.033	0.24	0.59	0.80
δ-decalactone	0.09	0.10	0.13	0.009	0.10	0.16	0.67
Sum, lactones	0.45	0.55	0.38	0.033	0.56	0.22	0.42

GT = dairy cows adapted to fresh herbage feeding before grazing; AT = dairy cows not adapted to fresh herbage feeding before grazing; IND = dairy cows kept in cubicle system; SEM = standard error of the mean; AM: adaptation method; D = day of sampling; ^a,b^: means statistically different.

**Table 3:** Mean values of variables related to feeding and locomotion behavior (*n* = 18).

Item	Adaptation Method			SEM	*p*-Value		
	GT	AT	IND		AM	D	AM × D
Eating time (min/d)	444.8	503.4	421.1	17.20	0.14	0.03	0.67
Eating chews (n/d)	31,188	34,876	30,919	1392.5	0.47	0.01	0.44
Rumination time (min/d)	370.1 ^b^	334.9 ^b^	520.6 ^a^	9.39	<0.01	<0.01	0.21
Rumination chews (n/d)	23,089 ^b^	21,001 ^b^	30,017 ^a^	1104.2	0.01	<0.01	0.34
Boluses (n/d)	402.3 ^b^	394.0 ^b^	494.1 ^a^	14.41	0.01	<0.01	0.74
Lying time (min/d)	588.7	582.4	670.5	18.73	0.13	<0.01	0.08
Walking time (min/d)	135.0 ^a^	143.7 ^a^	48.6 ^b^	3.62	<0.01	<0.01	<0.01
Standing time (min/d)	716.7	714.3	721.3	17.41	0.93	<0.01	0.34
Steps (n/d)	3894 ^a^	4047 ^a^	1104 ^b^	105.5	<0.01	<0.01	<0.01

GT = dairy cows adapted to fresh herbage feeding before grazing; AT = dairy cows not adapted to fresh herbage feeding before grazing; IND = dairy cows kept in cubicle system; SEM = standard error of the mean; AM = adaptation method; D = day of measurements; ^a,b^: means statistically different.
